# Establishment and Validation of a CUMS Model for Depression-like Behavior in Honeybees: A Tool for Pollinator Stress Research

**DOI:** 10.3390/insects17080773

**Published:** 2026-07-27

**Authors:** Yunqi Yang, Shuying Dai, Lizhen Zhang

**Affiliations:** 1 Honeybee Research Institute, Jiangxi Agricultural University, Nanchang 330045, China; yangyunqi@stu.jxau.edu.cn (Y.Y.); dsy1227@126.com (S.D.); 2 Jiangxi Province Key Laboratory of Honeybee Biology and Beekeeping, Jiangxi Agricultural University, Nanchang 330045, China

**Keywords:** chronic unpredictable mild stress (CUMS), depression, *Apis mellifera*, animal models

## Abstract

Honeybees (*Apis mellifera*), as key pollinating insects, are undergoing population decline, which has become a critical environmental challenge affecting ecosystem stability and sustainable agriculture. Moreover, a potential association exists between the physiological and behavioral responses of honeybees induced by environmental stress and the pathological mechanisms of depression. The pathogenesis of depression remains complex and not fully understood, and a stable animal model is a prerequisite for investigating its mechanisms and screening antidepressants. This study aimed to examine the alterations induced by CUMS and to evaluate behavioral changes related to depression-like phenotypes and neurotransmitter levels in the honeybee brain. The results showed that CUMS treatment induced typical depression-like behaviors and downregulated monoamine neurotransmitters in honeybees, consistent with observations in mammalian depression models. Fluoxetine was found to reverse these abnormalities, further supporting the validity of the model. This study provides a novel tool for depression research, antidepressant screening, and stress-related investigations in pollinators.

## Simple Summary

Honeybees play a critical role in pollination and ecosystem stability; however, environmental stress can induce depression-like behaviors in these insects. The establishment of a reliable animal model is essential for investigating depression and stress responses in pollinators. In this study, a chronic unpredictable mild stress (CUMS) model was established and validated in honeybees. Experimental results showed that stressed honeybees exhibited decreased spontaneous activity, reduced sucrose preference, prolonged proboscis extension response latency, and increased sting extension response, along with altered levels of neurotransmitters such as octopamine and dopamine in the brain. Notably, fluoxetine did not disrupt neurotransmitter homeostasis in normal honeybees and partially restored the neurotransmitter imbalance induced by CUMS. This honeybee depression model provides a novel approach for examining the effects of stress on pollinator health and serves as a useful tool for antidepressant screening and studies of stress mechanisms.

## 1. Introduction

Depression is a highly prevalent mental disorder that markedly affects both physical and psychological well-being [1]. It has been reported that around 17% of people experience depression at least once during their lifetime [2]. According to the World Health Organization (WHO) [3], depression affects about 332 million people worldwide. Stressful life events are associated with a five-fold increase in the risk of developing depression, identifying stress as one of the most important external causes of this affective disorder [4]. Therefore, depression is not a “one disease” state, but rather represents a spectrum of disorders with overlapping or shared symptoms [5]. It is the leading cause of disability worldwide and imposes a substantial emotional and financial burden [6,7]. The proportion of patients with depression may continue to increase in the future, indicating the need for greater attention and further research on depression.

In mammalian systems, rodent models have provided fundamental insights into depression research [8,9]. Chronic unpredictable mild stress (CUMS) is a widely used stress-induced rodent model of depression, in which animals are repeatedly exposed to a variety of mild and unpredictable physical and psychological stressors over a prolonged period, thereby inducing depression-like phenotypes, including anhedonia, behavioral despair, cognitive impairment, and sleep-related disturbances [10,11,12]. However, rodent models require substantial financial investment [13]. The zebrafish (*Danio rerio*) has emerged as a novel vertebrate model [14], possessing strong genetic manipulability and the ability to exhibit various depression-related behavioral phenotypes [15], such as behavioral despair [16], anhedonia [17], and anxiety-like behaviors [18]. Nevertheless, zebrafish models are limited by difficulties in standardizing behavioral phenotypes [19] and comprehensively annotating neural circuits [20]. Among invertebrate models, *Drosophila melanogaster* (*D. melanogaster*) has become an important model for investigating the molecular and neural circuit mechanisms underlying depression because of its low cost, short life cycle, rapid reproduction, and advanced genetic tools [21,22]. In contrast to *Drosophila*, honeybees exhibit complex multilevel social traits, including colony labor division, dance communication, and group collaboration [23]. Upon exposure to aversive stimuli, honeybees display fear-like behavioral responses that recapitulate human fear-related phenotypes. Furthermore, honeybees exhibit stress-related changes in monoamine signaling, while dopamine regulates food reward and feeding motivation through evolutionarily conserved neural mechanisms [24,25]. Thus, honeybees represent a suitable model for biomedical studies of stress and emotional disorder mechanisms.

Honeybees are social insects that experience considerable survival pressures under natural conditions, including climate change, environmental pollution, pathogen and pest threats, and food scarcity [24,26]. In this context, well-characterized behavioral responses, such as the proboscis extension response (PER) and sting extension response (SER), are commonly used in honeybee stress research. The PER is an innate appetitive feeding reflex elicited by stimulation of the antennae or mouthparts with sucrose solution, and it is widely used to assess reward-related behavioral plasticity in insects [27]. Similarly, the SER reflects defensive responses triggered by aversive stimuli in honeybees. Aversion is an emotional characteristic of animal depression, and aggression is an external manifestation of certain depressive-like mental disorders [28]. Thus, it is feasible to establish a method for evaluating depressive-like behaviors based on the natural behavioral responses of honeybees. Furthermore, a series of behavioral studies in honeybees has demonstrated that honeybees can exhibit emotions analogous to those observed in humans [24]. For example, after intense shaking that simulates predator attacks, honeybees display pessimistic cognitive bias and show negative avoidance toward ambiguous odor stimuli [24]. In contrast, when rewarded with sweet food, dopamine levels associated with positive emotions increase in the honeybee brain, accompanied by optimistic and positive emotion-like behaviors [24]. Therefore, honeybees possess the emotional basis for developing depressive-like states. Taken together, the consistency of stressors and behavioral evaluation criteria supports the feasibility of establishing a CUMS depression model in honeybees.

This study aimed to establish honeybees (*Apis mellifera*) as a model for investigating CUMS-induced alterations associated with depressive-like behavior and assessing brain neurotransmitter levels. This model focuses on the species-specific characteristics of honeybees as social insects and incorporates stress-induced changes in social behavior and dynamic variations in neurotransmitters into the evaluation framework. The study showed that CUMS altered honeybee behaviors, including spontaneous activity level, sucrose preference, PER latency, and defensive response, and suppressed the levels of several brain neurotransmitters such as dopamine, octopamine, and 5-HT. After treatment with the antidepressant fluoxetine (FLX), partial behavioral indicators of honeybees, including spontaneous activity, sucrose preference, and defensive response, were significantly restored. The establishment of the honeybee CUMS model of depression-like behavior provides a novel invertebrate paradigm for depression research.

## 2. Materials and Methods

### 2.1. Materials and Experimental Animals

Honeybees (*Apis mellifera*) used in this study were supplied by the experimental apiary of the Honeybee Research Institute, Jiangxi Agricultural University, Nanchang, China (28.77° N, 115.83° E). Newly emerged honeybees used in all experiments were obtained from brood frames collected from healthy experimental hives within the same apiary and maintained in a constant-temperature and humidity incubator (DHI-150S, AIKANE, Shanghai, China; T: 35 °C, RH: 65%). To reduce the diversity of the gut microbiota and obtain conventional honeybees, the bees were fed 50% (*w*/*v*) sucrose syrup prepared from sucrose (Xilong Scientific, Shantou, China) on the first day. Subsequently, honeybees were fed homogenates prepared from freshly dissected hindguts of nurse honeybees from their hives of origin, mixed with 1 mL 1× PBS and 1.5 mL 50% (*w*/*v*) sucrose syrup for 24 h. Thereafter, honeybees were fed a mixture of pollen and 50% (*w*/*v*) sucrose syrup (1:1) until tissue collection.

### 2.2. Methods

#### 2.2.1. CUMS

In this study, the protocol for chronic unpredictable mild stress (CUMS) was modified based on previously reported stress-induced depression-like models in insects [29] to establish a depression-like model in honeybees. Newly emerged honeybees were randomly assigned to nine experimental groups, including eight CUMS treatment groups (CUMS-1–CUMS-8) and one untreated blank control group without stress exposure throughout the experiment. Each group contained 60 individual honeybees, resulting in 540 bees per independent experiment, and all experimental procedures were performed with six biological replicates. Following one day of acclimation, honeybees in the CUMS groups were subjected to an 8-day chronic stress paradigm comprising four stressor types: odor stress, agitation stress, electric stress, and starvation stress. The eight CUMS groups differed in stressor intensity and exposure sequence, as detailed in Table S1. All stress interventions were conducted at 9:00 a.m. daily.

**Figure 1:**
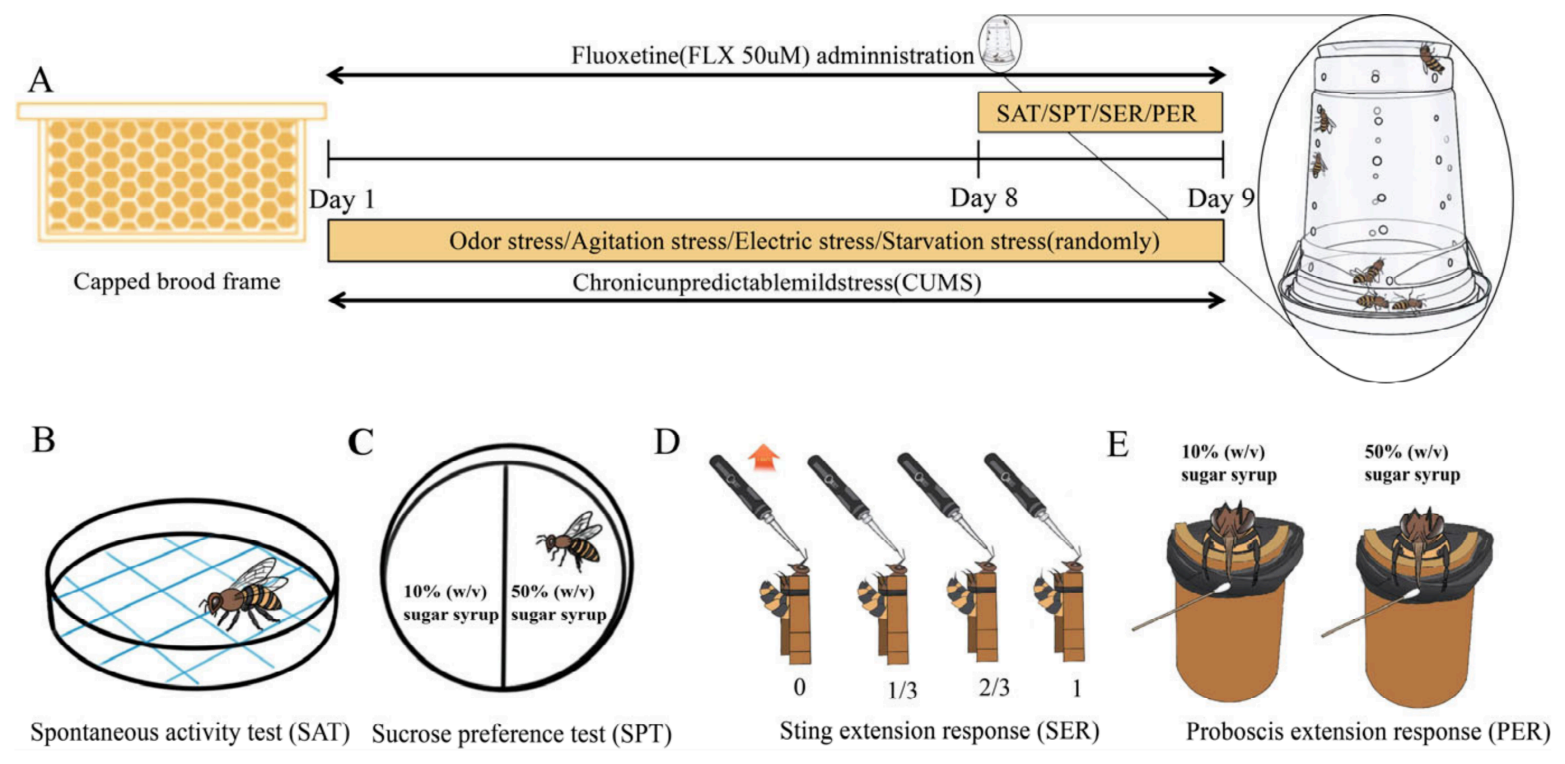
Schematic representation of the experimental protocol. (**A**) Experimental timeline of CUMS modeling and FLX intervention; FLX 50 μM administration was included only in the FLX intervention experiment. (**B**–**E**) Schematic diagrams of honeybee behavior. SAT: spontaneous activity test, SPT: sucrose preference test, SER: sting extension response, PER: proboscis extension response. In panel (**D**), the red arrow indicates that the copper thermal probe was heated to 60 °C before stimulation.

Before each daily stress treatment, dead honeybees in each group were counted and removed as commonly performed in caged honeybee survival studies [30]. At 24 h after stress treatment, samples were randomly collected from each group. Eight honeybees per group were used for behavioral tests, including the spontaneous activity test (SAT), sucrose preference test (SPT), sting extension response (SER), and proboscis extension response (PER). Meanwhile, additional honeybees were snap-frozen in liquid nitrogen for determination of brain neurotransmitter levels, including six core neurotransmitters: histamine (HA), octopamine (OA), dopamine (DA), 5-hydroxytrypta-mine (5-HT), γ-aminobutyric acid (GABA), and L-glutamate (L-Glu). The schematic representation of the experimental protocol is shown in Figure 1.

Notably, standardized stress intervention rules were implemented during the 8-day CUMS modeling period. Specifically, one stressor was randomly selected each day and applied twice consecutively, with no identical stressor administered on two successive days. To minimize potential confounding effects of acute starvation on feeding-related behavioral tests, starvation stress was excluded on the final day of CUMS treatment before the SPT and PER assays.

#### 2.2.2. Stress Test

The experiment employed odor stress, shock stress, electric stress, and fasting stress to perform chronic mild stress treatment on honeybees: (1) Odor stress: Isoamyl acetate is a major component of bee alarm pheromones and can induce stress responses in both young and foraging honeybees. Isoamyl acetate (Macklin, Shanghai, China) was diluted to 8% or 24% with mineral oil (Macklin, Shanghai, China). Then, 48 μL was adsorbed onto a filter paper with a diameter of 3 cm, after which the filter paper and honeybees were placed in a closed plastic cup for stress treatment for 10 min [31]. (2) Agitation stress: Based on the oscillation stress conditions previously used in fruit flies, honeybees were placed in a 50 mL centrifuge tube and transferred onto a vibration device (SA-JZ, Wuxi Shiao Technology, Wuxi, China). Vibration at 200 Hz was applied for 20 s, followed by a 10 s pause to minimize short-term habituation or immediate adaptation within a single stimulation session. The 20 s/10 s cycle was repeated for 15 min, and two agitation sessions were conducted with a 30 min interval between sessions [32]. (3) Electric stress: honeybees were transferred into narrow tube enclosures with conductive meshes on the inner surfaces. Subsequently, honeybees were exposed to mild electric stimuli of either 5 V or 7 V through the power grid for 1 min intervals, three times daily, with 4 h intervals between sessions. (4) Starvation stress: honeybees were subjected to 2 h or 4 h of food deprivation.

#### 2.2.3. Spontaneous Activity Test (SAT)

The spontaneous activity test was performed according to the methodology described in the previously published study by Humphries et al. [33]. In this procedure, a single bee was introduced into a clean Petri dish with a grid pattern drawn on the bottom. The grid consisted of evenly spaced lines, each 2 cm apart, thereby forming squares with side lengths of 2 cm. After the bee was placed inside the dish, its movement was observed for a standardized duration of two minutes. During this period, the number of grid squares crossed by the bee was carefully recorded. The level of spontaneous activity was quantified by calculating the total number of grid crossings per unit time, providing a measurable indicator of the bee’s locomotor activity (Figure 1B).

#### 2.2.4. Sucrose Preference Test (SPT)

The sucrose preference test was performed according to a previously published protocol by Ramos-Hryb et al. [34]. A piece of filter paper was accurately cut to fit tightly into a clean Petri dish. One half of the filter paper was fully saturated with a 10% (*w*/*v*) sucrose syrup solution, whereas the other half was saturated with a 50% (*w*/*v*) sucrose syrup solution. A single honeybee was gently introduced into the center of the Petri dish, along the boundary between the two sucrose-treated filter paper areas, such that its initial position was approximately equidistant from the 10% and 50% sucrose sides. The behavior of the honeybee was observed for a standardized duration of one minute. During this period, the time spent by the bee in proximity to each of the two sucrose concentrations was carefully recorded. The sucrose preference index (PI) was calculated using the formula: PI = (T50 − T10)/60 s, where T50 represents the time (in seconds) spent on the side containing the 50% sucrose solution, and T10 represents the time (in seconds) spent on the side containing the 10% sucrose solution. This preference index provides a quantitative indicator for evaluating whether changes in food preference occur in response to different sucrose concentrations (Figure 1C).

#### 2.2.5. Sting Extension Response (SER)

The SER of honeybees was conducted based on the method previously reported by Lenoir et al. [35]. Honeybees were restrained within copper tubes and subjected to a 60 min acclimatization period. A copper thermal probe (Wuxi Shiao Technology, Wuxi, China) was heated to 60 °C and immediately brought into contact with the antenna of honeybees for 1 s. Subsequently, the intensity of sting extension and the extent of the response in honeybees after the 1-sthermal stimulation were assessed via real-time live observation. A scoring criterion for the behavioral test was then established according to the methodology described by Lenoir et al. [35]. A scoring system ranging from 1 to 4 was used to quantify the SER after thermal stimulation: a score of 1 was assigned for abdominal curling without sting extension; 2 for abdominal curling with extension of one-third of the sting; 3 for abdominal curling with extension of two-thirds of the sting; and a maximum score of 4 for vigorous abdominal contraction with full sting extension (Figure 1D). Standard reference photographs illustrating the four sting-extension grades were placed beside the test platform to assist in distinguishing different response levels and to reduce subjective bias. The observer was blinded to the treatment groups throughout the experiment. Honeybees were kept in coded copper tubes that concealed their treatment information, and group identities were decoded only after all scoring had been completed. Before the formal experiment, the observer received standardized training using the reference photographs. In addition, 30 randomly selected honeybees were independently scored by two observers. The intraclass correlation coefficient (ICC) exceeded 0.91, indicating excellent agreement between observers. During testing, individual honeybees were positioned near an exhaust fan to prevent the accumulation of alarm pheromones, which could interfere with subsequent tests. The SER score was used as an indicator of the intensity of the individual defensive response.

#### 2.2.6. Proboscis Extension Response (PER)

The PER of honeybees was assessed according to the method described by Li et al. [36]. Honeybees were restrained in metal tubes and allowed a 60 min acclimation period before testing. Following acclimation, 10% and 30% sucrose solutions were presented to the honeybees. The solutions were carefully presented without touching the antennae to induce a PER based solely on olfactory cues. The latency of the PER (from solution presentation to full extension of the proboscis) was recorded within a 15 s observation window. If no PER occurred within this period, a latency of 15 s was assigned as the censored data point. Differences in PER latency were statistically analyzed as an indicator of foraging motivation in honeybees (Figure 1E).

#### 2.2.7. LC-MS Analysis

For each group, 24 honeybees were randomly selected from the six biological replicate cages, with all six cages represented, and their brains were dissected. Every three brains were pooled to form one sample, yielding eight samples per group for quantitative analysis of brain neurotransmitters. The dissected brain tissue was treated with 300 µL aqueous solution of dihydroxybenzylamine hydrobromide hydrochloride (Sigma-Aldrich, St. Louis, MO, USA) and homogenized at a speed of 6.5 m/s for 60 s using a tissue homogenizer (MP Biomedicals, Burlingame, CA, USA). The homogenate was centrifuged at 12,000× *g* for 5 min at 4 °C, followed by sonication at 0 °C for 3 min, and then centrifuged again at 12,000× *g* for 3 min. The supernatant was transferred into a 450 µL 0.22 µm hydrophilic PTFE filter vial (SCEQ-FV31022, ANPEL, Shanghai, China) to remove impurities, and the filtered liquid was transferred into sample vials for LC/MS analysis. The levels of OA, DA,5-HT, HA, L-Glu, and-GABA in samples were analyzed using the Agilent 6546 Triple Quadrupole LC/MS system (Agilent, Santa Clara, CA, USA). The chromatographic column used was Agilent Polaris C18-A (150 mm × 4.6 mm, i.d., 5 μm) (Part No. A2000150X046, Agilent, Santa Clara, CA, USA), with an injection volume of 10 µL and a flow rate of 0.5 mL/min. Eluent A consisted of 0.1% formic acid (≥99%, LC–MS grade, F809715, Macklin, Shanghai, China) in water, and eluent B consisted of methanol (≥99.9%, LC–MS grade, M813903, Macklin, Shanghai, China) The gradient elution program was established as follows: 0~1 min, 5% A; 1~15 min, 5~50% A; 15~15.5 min, 50% A; 15.50~28.00 min, 90% A; 28.00~35.00 min, 5% A. The mass spectrometry parameters were optimized as follows: sheath gas temperature, 275 °C; flow rate of sheath gas, 12 L/min; dry gas temperature, 325 °C; flow rate of dry gas, 9 L/min; nebulizer gas pressure, 310 kPa; fragmentor voltage, 90 V; capillary voltage, 4000 V. The external standard method was used, with dopamine hydrochloride (Sigma-Aldrich, Darmstadt, Germany), octopamine hydrochloride (Sigma-Aldrich, Darmstadt, Germany), serotonin hydrochloride (Sigma-Aldrich, Darmstadt, Germany), histamine (Vetec™, Sigma-Aldrich, St. Louis, MO, USA), L-glutamate (Sigma-Aldrich, Darmstadt, Germany), and γ-aminobutyric acid (Solarbio, Beijing, China) used as standard samples. Data collection and analysis were performed using MassHunter Quantitative Analysis software, version 10.1 (Agilent, Santa Clara, CA, USA). The peak areas of each compound were substituted into the corresponding standard curves prepared using authentic standards, as shown in Table S2, for quantitative analysis, and the contents of each neurotransmitter were calculated.

#### 2.2.8. FLX Treatment

Fluoxetine (FLX) is a classic antidepressant widely used in clinical practice. Therefore, FLX (343290, EMD Millipore Corp., Burlington, MA, USA) intervention was conducted in this study to investigate whether it could reverse CUMS-induced behavioral impairments and disturbances in brain neurotransmitter levels in honeybees. Newly emerged honeybees were collected from different source hives using the same sampling procedure as in the other experiments and were randomly assigned to four groups: Control, FLX 50 μM, CUMS, and CUMS + FLX 50 μM. Each group included six replicate cages, with 60 honeybees per cage, giving a total of 360 honeybees per group. Based on the results of behavioral assays and brain neurotransmitter measurements, CUMS-3 was selected as the final CUMS protocol for fluoxetine validation experiments. Honeybees in the CUMS and CUMS + FLX 50 μM groups were exposed to odor stress (24% concentration), agitation, electric stress (voltage: 5 V), and food deprivation (duration: 2 h). The exposure period lasted for 8 days, and each stressor was administered twice, with no identical stressor applied on two consecutive days. Honeybees in the Control group were not subjected to stress throughout the experimental period, consistent with Section 2.2.1. Honeybees in the FLX 50 μM group received the same treatment as the Control group, except that 50 μM fluoxetine (FLX) was added to the daily sucrose-pollen feeding solution. The CUMS + FLX 50 μM group underwent the same CUMS procedure as the CUMS group, with the addition of 50 μM FLX in the daily sucrose-pollen diet. In the FLX intervention experiment, drug administration lasted for 9 days (Figure 1). Samples were collected 24 h after completion of all stress treatments for subsequent behavioral testing and quantitative analysis of brain neurotransmitters. Detailed protocols for sample collection and measurements are provided in Section 2.2.2, Section 2.2.3, Section 2.2.4, Section 2.2.5, Section 2.2.6 and Section 2.2.7.

#### 2.2.9. Statistical Analysis

All statistical analyses were performed using IBM SPSS Statistics 26 (https://www.ibm.com/cn-zh/spss, accessed on 18 July 2026). Data normality was assessed using the Shapiro–Wilk test. During CUMS protocol screening, mortality was recorded throughout the experiment, and survival rate was calculated as: survival rate (%) = (initial total number of honeybees − cumulative number of deaths)/initial total number of honeybees × 100%. Survival curves were estimated using the Kaplan–Meier method and compared using the log-rank test. For comparisons among the Control and CUMS-1 to CUMS-8 groups, SAT and SPT data were analyzed using Welch’s one-way ANOVA followed by pairwise Welch’s *t*-tests, whereas SER and PER data were analyzed using the Kruskal–Wallis test followed by pairwise Wilcoxon rank-sum tests. For prespecified comparisons between CUMS protocols differing only in odor, electric, or starvation stress intensity, SAT, SPT, and PER data were analyzed using one-way ANOVA with pairwise contrasts, SER data using a quasibinomial generalized linear model with pairwise contrasts, and neurotransmitter data using Welch’s *t*-tests or Wilcoxon rank-sum tests according to normality.

For fluoxetine experiments, one-way ANOVA or Welch’s one-way ANOVA was applied to normally distributed multi-group data, and Student’s or Welch’s *t*-tests were used for subsequent pairwise comparisons depending on variance homogeneity. For non-normally distributed data, Kruskal–Wallis tests were performed, followed by Wilcoxon rank-sum tests. Two-group comparisons were conducted using Student’s *t*-test, Welch’s *t*-test, or the Wilcoxon rank-sum test, as appropriate. Pairwise *p* values were adjusted using the Holm method, except for prespecified stressor-intensity comparisons, for which unadjusted *p* values were reported. Honeybee movement trajectories were visualized using ImageJ 1.54s (https://imagej.net/ij/download.html, accessed on 10 June 2026). Data are presented as mean ± SEM unless otherwise stated. Statistical significance was defined as * *p* < 0.05, ** *p* < 0.01, and *** *p* < 0.001.

## 3. Results

### 3.1. Honeybees Show Sensitivity in Behavioral Tests Under CUMS

**Figure 2:**
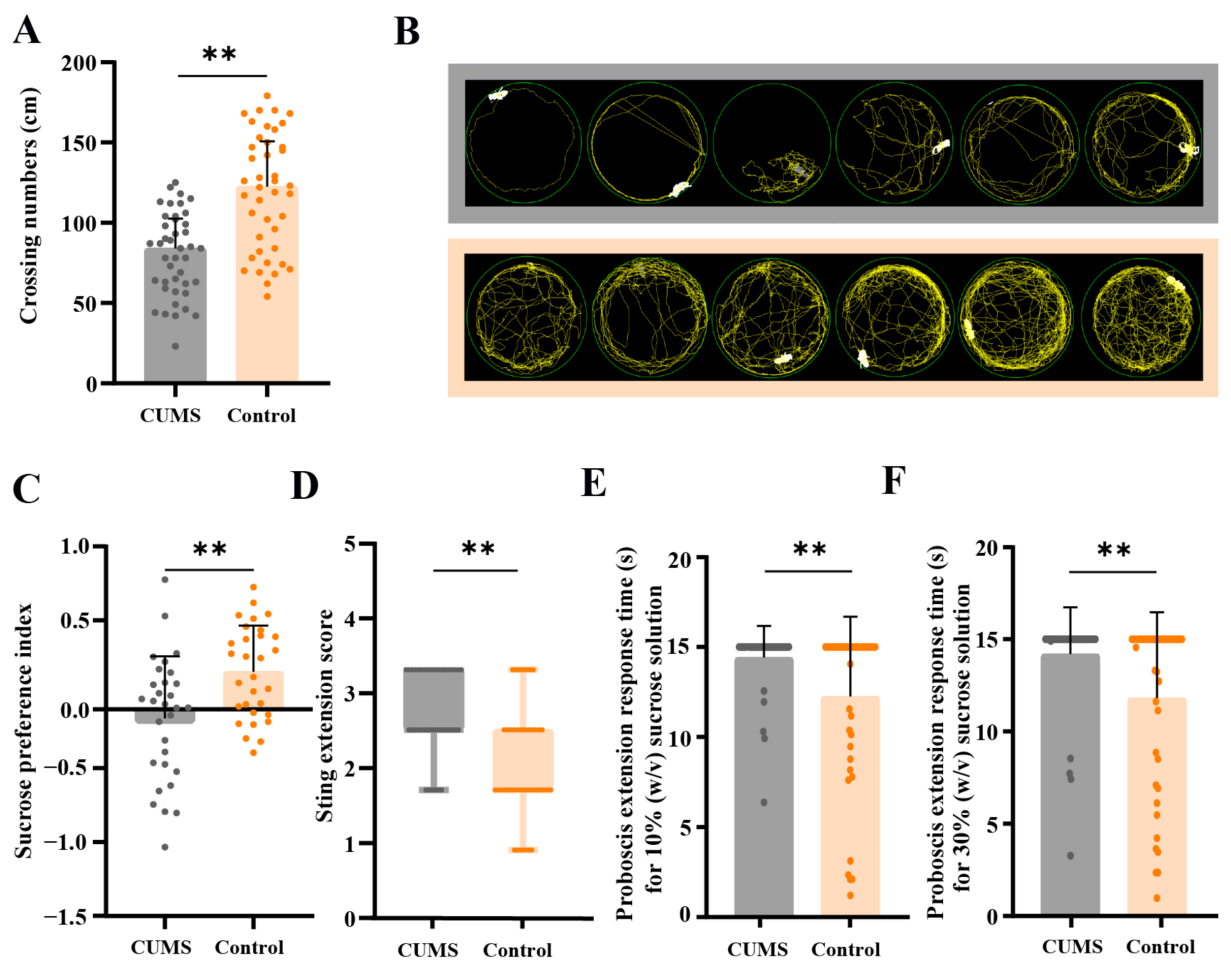
Behavioral assays of honeybees after CUMS treatment. (**A**) SAT results in Control (*n* = 42) and CUMS (*n* = 42) honeybees. CUMS significantly reduced spontaneous activity compared with Control (*W* = 1406.5, *p* < 0.01). (**B**) Representative movement trajectories of honeybees in the CUMS (*n* = 42) and Control *(n* = 42) groups. Grey denotes the CUMS group, whereas light orange denotes the Control group. (**C**) SPT results in Control *(n* = 30) and CUMS (*n* = 30) honeybees. CUMS significantly reduced sucrose preference compared with Control (*t* = 3.298, df = 58, *p* < 0.01). (**D**) SER results in Control (*n* = 42) and CUMS (*n* = 42) honeybees. CUMS significantly increased SER compared with Control (*W* = 452.5, *p* < 0.01). (**E**,**F**) PER latency tested with 10% and 30% (*w*/*v*) sucrose solutions, respectively, in Control (*n* = 42) and CUMS (*n* = 42) honeybees. The PER latency was recorded with a 15 s cut-off time. CUMS significantly increased PER latency at both 10% sucrose (*W* = 655.5, *p* < 0.01) and 30% sucrose (*W* = 602.0, *p* < 0.01). Bars represent the mean ± SEM, with individual data points shown. Box plots show the median and interquartile range, with whiskers indicating the range. CUMS: chronic unpredictable mild stress; Control: no CUMS; SAT: spontaneous activity test; SPT: sucrose preference test; PER: proboscis extension response test; SER: sting extension response test. ** *p* < 0.01 compared with the Control group.

Eight CUMS protocols (Table S1) were used to evaluate the susceptibility of honeybees to CUMS after 8 days, as outlined in the experimental workflow shown in Figure 1. The spontaneous activity of honeybees (i.e., the number of grid crossings within 2 min) was significantly lower in all CUMS treatment groups than in the Control group (Figure S1, |*t*| = 4.15–7.82, df = 74.12–81.73; Figure 2A, *W* = 1406.5, *p* < 0.05). Video tracking of honeybee locomotion indicated that individuals subjected to CUMS exhibited confined activity within small zones and reduced exploration of the central region (Figure 2B). Using the sucrose preference test to assess reward responsiveness, it was observed that, compared with the Control group, the preference index (PI) scores of honeybees in the CUMS groups were significantly reduced (Figure S2 |*t*| = 3.10–7.22, df = 46.98–57.97, *p* < 0.01; Figure 2C, *t* = 3.298, df = 58, *p* < 0.01). Standardized SER testing was used to evaluate changes in defensive behavior. The results showed that honeybees in the CUMS groups, except for CUMS-2 and CUMS-4, exhibited increased sting extension scores (Figure S3, *W* = 1199.5–1311.5; Figure 2D, *W* = 452.5, *p* < 0.01). In addition, the PER induced by 10% (*w*/*v*) and 30% (*w*/*v*) sucrose solutions was significantly prolonged, except in CUMS-6 and CUMS-7 (Figure S4 and Figure 2E,F; significant comparisons: *U* = 1028.0–1194.0, all *p* < 0.05; CUMS-6 and CUMS-7 at 30% sucrose: *U* = 1036.0–1068.0, both *p* > 0.05). Compared with the Control group, all eight stress protocols significantly reduced honeybee survival rates (Figure S5; χ^2^ = 14.18–196.93, df = 1, all *p* < 0.01). Among these, honeybees exposed to the CUMS-3 condition exhibited the highest survival rate (81.94%; χ^2^ = 14.18, *p* < 0.01), whereas those subjected to CUMS-7 showed the lowest survival rate (43.06%; χ^2^ = 196.93, *p* < 0.01) (Figure S5).

### 3.2. CUMS Inhibits Certain Neurotransmitter Levels in Honeybee Brains

**Figure 3:**
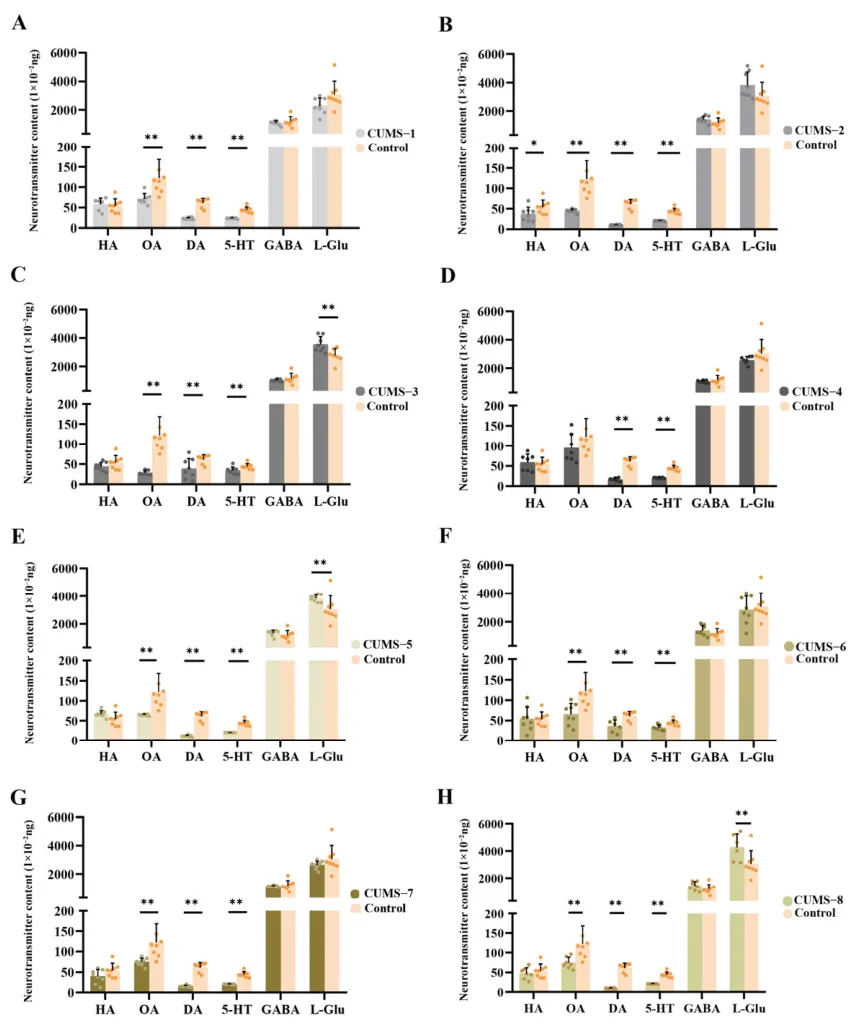
Neurotransmitter levels in the brains of honeybees under different CUMS protocols. Brain levels of HA, OA, DA, 5-HT, GABA, and L-Glu are quantified by LC–MS following exposure to eight CUMS protocols. (**A**) Compared with the Control group, CUMS-1 significantly reduced OA (*t* = 3.113, df = 14, *p* < 0.01), DA (*W* = 56, *p* < 0.01), and 5-HT levels (*t* = 8.372, df = 14, *p* < 0.01). (**B**) CUMS-2 significantly reduced HA (*t* = −2.261, df = 14, *p* < 0.05), OA (*t* = 4.863, df = 14, *p* < 0.01), DA (*W* = 56, *p* < 0.01), and 5-HT levels (*W* = 64, *p* < 0.01). (**C**) CUMS-3 significantly reduced OA (*t* = 5.842, df = 14, *p* < 0.01), DA (*W* = 45, *p* < 0.01), and 5-HT levels (*t* = 3.010, df = 14, *p* < 0.01), while increasing L-Glu levels (*t* = −3.096, df = 14, *p* < 0.01). (**D**) CUMS-4 significantly reduced DA (*W* = 49, *p* < 0.01) and 5-HT levels (*t* = 8.910, df = 13, *p* < 0.01). (**E**) CUMS-5 significantly reduced OA (*t* = 3.645, df = 7.03, *p* < 0.01), DA (*W* = 56, *p* < 0.01), and 5-HT levels (*t* = 9.859, df = 7.01, *p* < 0.01), while increasing L-Glu levels (*t* = −3.215, df = 14, *p* < 0.01). (**F**) CUMS-6 significantly reduced OA (*t* = 3.080, df = 14, *p* < 0.01), DA (*W* = 39, *p* < 0.01), and 5-HT levels (*t* = 3.432, df = 14, *p* < 0.01). (**G**) CUMS-7 significantly reduced OA (*t* = 2.985, df = 14, *p* < 0.01), DA (*W* = 56, *p* < 0.01), and 5-HT levels (*t* = 9.611, df = 14, *p* < 0.01). (**H**) CUMS-8 significantly reduced OA (*t* = 3.025, df = 13, *p* < 0.01), DA (*W* = 49, *p* < 0.01), and 5-HT levels (*t* = 8.886, df = 13, *p* < 0.01), while increasing L-Glu levels (*t* = −3.187, df = 12, *p* < 0.01). Statistical significance was defined as * *p* < 0.05, ** *p* < 0.01.

Following CUMS exposure, both DA (*W* = 39–56, all *p* < 0.01) and 5-HT levels (|*t*| = 3.010–9.859, df = 7.01–14, or *W* = 64; all *p* < 0.01) were significantly reduced under all eight protocols (Figure 3A–H). OA levels were significantly reduced in all groups except CUMS-4 (|*t*| = 2.985–5.842, df = 7.03–14, all *p* < 0.01). L-Glu levels were significantly increased in CUMS-3, CUMS-5, and CUMS-8 (Figure 3C,E,H; |*t*| = 3.096–3.215, df = 12–14, all *p* < 0.01), whereas HA was significantly reduced only in CUMS-2 (Figure 3B; *t* = −2.261, df = 14, *p* < 0.05). Overall, CUMS of varying intensities disrupted the homeostasis of OA, DA, and 5-HT in the honeybee brain. These monoamine neurotransmitters serve as key physiological indicators for evaluating depression-like phenotypes, and their alteration patterns are consistent with the pathological characteristics observed in depression models of other animal species.

### 3.3. Effects of Different Stress Intensities on Behavioral Phenotypes and Brain Neurotransmitter Levels in Honeybees

**Figure 4:**
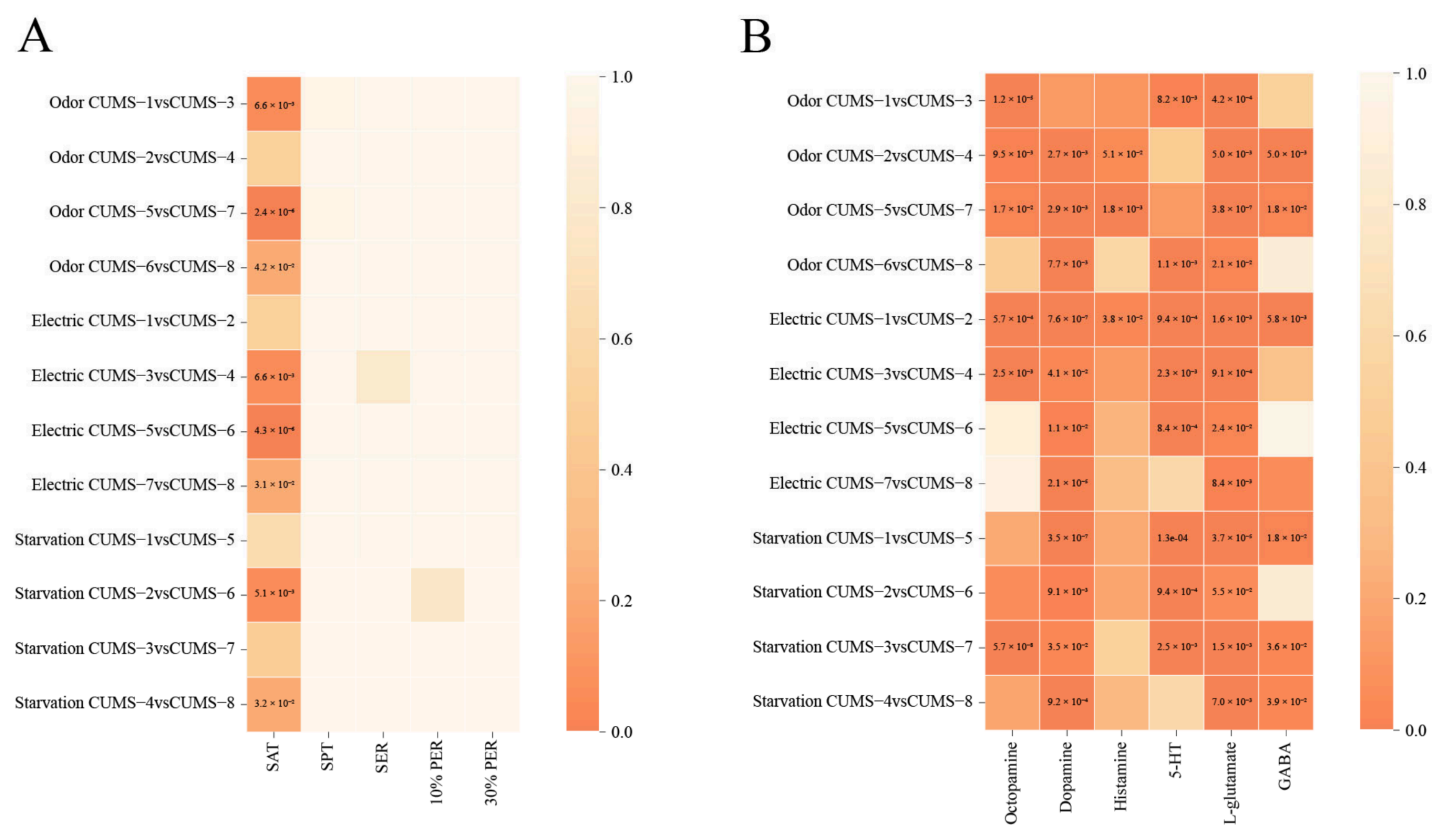
Pairwise comparison heatmaps based on behavioral and neurotransmitter *p* values across CUMS protocols. (**A**) Heatmap generated from pairwise comparison *p* values for SAT, SPT, SER, 10% PER, and 30% PER. SAT, SPT, and PER data were analyzed using one-way ANOVA followed by prespecified pairwise contrasts, whereas SER data were analyzed using a quasibinomial generalized linear model followed by prespecified pairwise contrasts. (**B**) Heatmap generated from pairwise comparison *p* values for OA, DA, HA, 5-HT, L-Glu, and GABA. For each neurotransmitter comparison, Welch’s *t*-test was used when both groups met the normality assumption, whereas the Wilcoxon rank-sum test was applied when either group did not meet the normality assumption. Pairwise comparisons were performed between CUMS protocols differing only in the intensity of a single stressor, including odor, electric, or starvation stress. Numbers within the cells and the color scales represent unadjusted *p* values, with darker colors indicating smaller *p* values. SAT: spontaneous activity test; SPT: sucrose preference test; PER: proboscis extension response test; SER: sting extension response test. OA: octopamine; DA: dopamine; 5-HT: serotonin; HA: histamine; L-Glu: L-glutamate; GABA: γ-aminobutyric acid.

After 8 days of CUMS exposure, honeybee behavioral phenotypes and brain neurotransmitter levels were analyzed to assess the effects of different stress intensities (Figure 4). Behavioral analysis revealed significant differences in SAT among the CUMS treatment groups (*F* = 7.13, *p* < 0.001), whereas no significant differences were detected in SPT, SER, or PER latency at either 10% or 30% sucrose (Figure 4A; *F* = 0.72–1.61, all *p* > 0.05). Significant differences were detected in HA, OA, DA, 5-HT, and L-Glu (Figure 4B, |*t*| = 3.59–13.19 or *W* = 0–64, all *p* < 0.05), whereas GABA did not differ significantly among the compared groups (Figure 4B, |*t*| = 0.18–3.66 or *W* = 9–55, all *p* > 0.05). Changes in SAT and neurotransmitter levels did not follow a consistent stress intensity-dependent trend. (Figure 4A,B).

Since behavioral and neurochemical alterations did not exhibit a consistent stress-intensity-dependent pattern, stressor intensity was not adopted as the primary criterion for screening CUMS regimens. Given that the CUMS model was designed to induce mild stress with minimal mortality, honeybee survival was also taken into account. Accordingly, CUMS-3 was selected as the optimal modeling protocol and is hereafter referred to as CUMS.

### 3.4. Fluoxetine Administration Improves Behaviors in Honeybees

**Figure 5:**
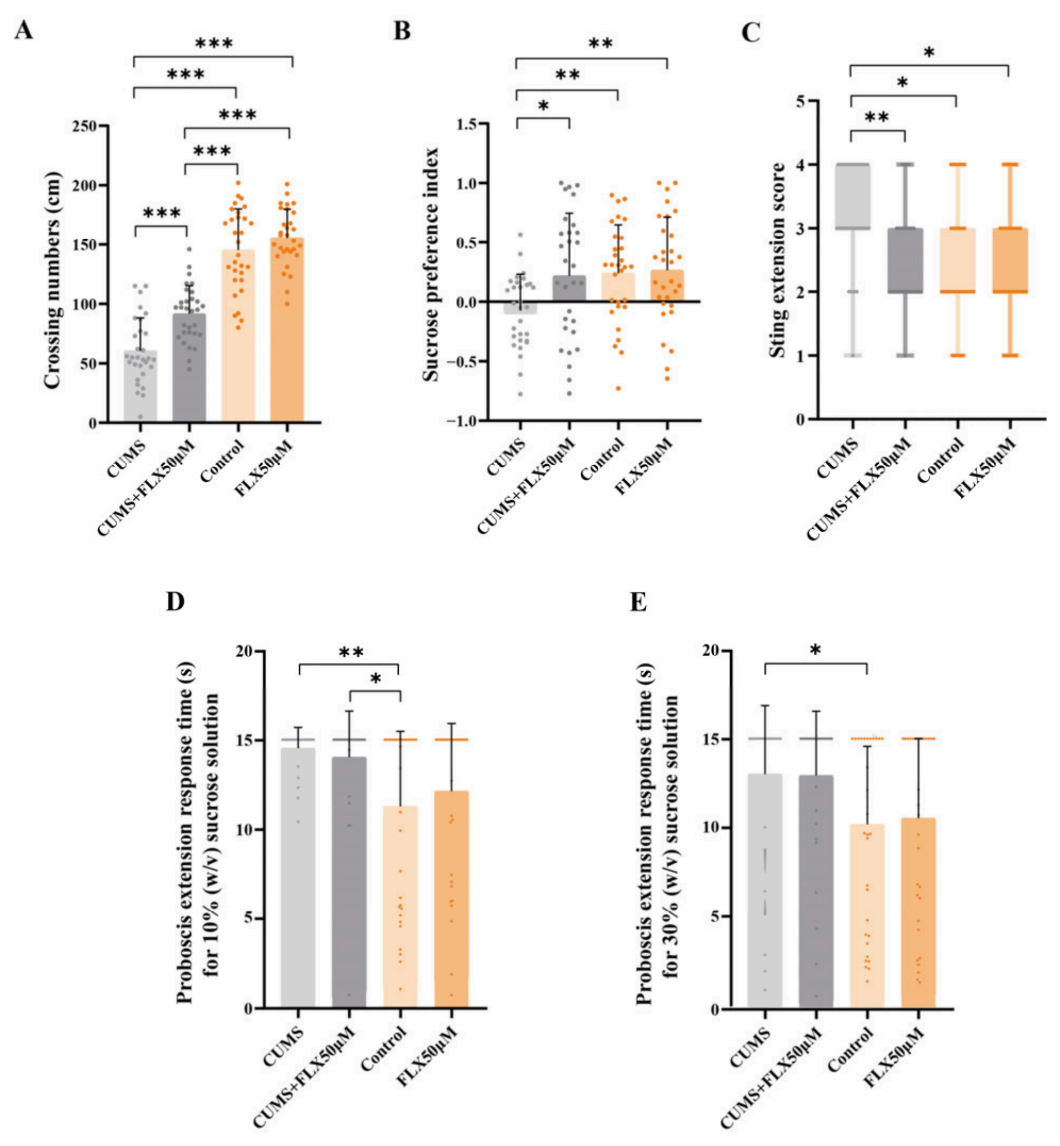
Behavioral changes in honeybees under four experimental treatments. (**A**) SAT of honeybees within 2 min (*n* = 30). CUMS reduced spontaneous activity, and CUMS + FLX 50 μM partially restored it (Control vs. CUMS, *t* = 10.46, df = 55.26, *p* < 0.001; CUMS vs. CUMS + FLX 50 μM, *t* = −4.70, df = 56.54, *p* < 0.001; Control vs. CUMS + FLX 50 μM, *t* = 7.01, df = 51.01, *p* < 0.001). (**B**) SPT in honeybees (*n* = 30). CUMS reduced sucrose preference, and CUMS + FLX 50 μM restored it (Control vs. CUMS, *t* = 3.36, df = 53.68, *p* < 0.01; CUMS vs. CUMS + FLX 50 μM, *t* = −2.61, df = 46.43, *p* < 0.05; CUMS vs. FLX 50 μM, *t* = 3.12, df = 54.56, *p* < 0.01). (**C**) SER in honeybees following CUMS exposure (*n* = 30). CUMS increased SER, and CUMS + FLX 50 μM attenuated it (Control vs. CUMS, *U* = 239.5, *p* < 0.05; CUMS vs. CUMS + FLX 50 μM, *U* = 637.0, *p* < 0.01; CUMS vs. FLX 50 μM, *U* = 401.5, *p* < 0.05). (**D**,**E**) PER latency assessed using 10% and 30% (*w*/*v*) sucrose solutions, respectively (*n* = 30). Significant pairwise comparisons were as follows: for 10% sucrose, Control vs. CUMS (*U* = 240.0, *p* < 0.01), Control vs. CUMS + FLX 50 μM (*U* = 264.0, *p* < 0.05); for 30% sucrose, Control vs. CUMS (*U* = 266.5, *p* < 0.05). FLX 50 μM alone had no significant effect compared with the Control group. Bars represent the mean ± SEM; box plots show the median and interquartile range, with whiskers indicating the range. * *p* < 0.05, ** *p* < 0.01, *** *p* < 0.001. CUMS: chronic unpredictable mild stress; Control: no CUMS; CUMS + FLX 50 μM: CUMS with 50 μM fluoxetine treatment; FLX 50 μM: 50 μM fluoxetine treatment without CUMS.

Fluoxetine administration during CUMS significantly improved performance in the SAT (*t* = −4.70, df = 56.54, *p* < 0.001), yet this recovery failed to reach the level measured in the Control group (*t* = 7.01, df = 51.01, *p* < 0.001; Figure 5A). In CUMS-exposed honeybees treated with fluoxetine, sucrose preference (*t* = −2.61, df = 46.43, *p* < 0.05; Figure 5B) and defensive sting extension response (*U* = 637.0, *p* < 0.01; Figure 5C) were markedly ameliorated. Notably, no statistically significant differences in SPT and SER were detected between the CUMS + FLX group and the Control group (Figure 5B,C; *p* > 0.05), indicating that fluoxetine restored these two behavioral readouts nearly to normal baseline levels. Although PER latencies at 10% and 30% (*w*/*v*) sucrose concentrations did not differ significantly between the CUMS and CUMS + FLX 50 μM groups (10%: *U* = 457.0, *p* > 0.05); (30%: *U* = 454.5, *p* > 0.05; Figure 5D,E), a declining trend in PER latency was observed. Honeybees treated with 50 μM fluoxetine alone showed no significant differences in SAT or SPT (|*t*| = 0.23–1.35, df = 51.78–57.54, both *p* > 0.05), or in SER and PER (*U* = 372.0–431.5, all *p* > 0.05), compared with the Control group (Figure 5A–E). Collectively, fluoxetine produced robust restorative effects on SAT, SPT, and SER in stressed honeybees, with SPT and SER indices restored almost to baseline normal levels.

### 3.5. Fluoxetine Administration Alters Neurotransmitter Levels in the Honeybee Brain

**Figure 6:**
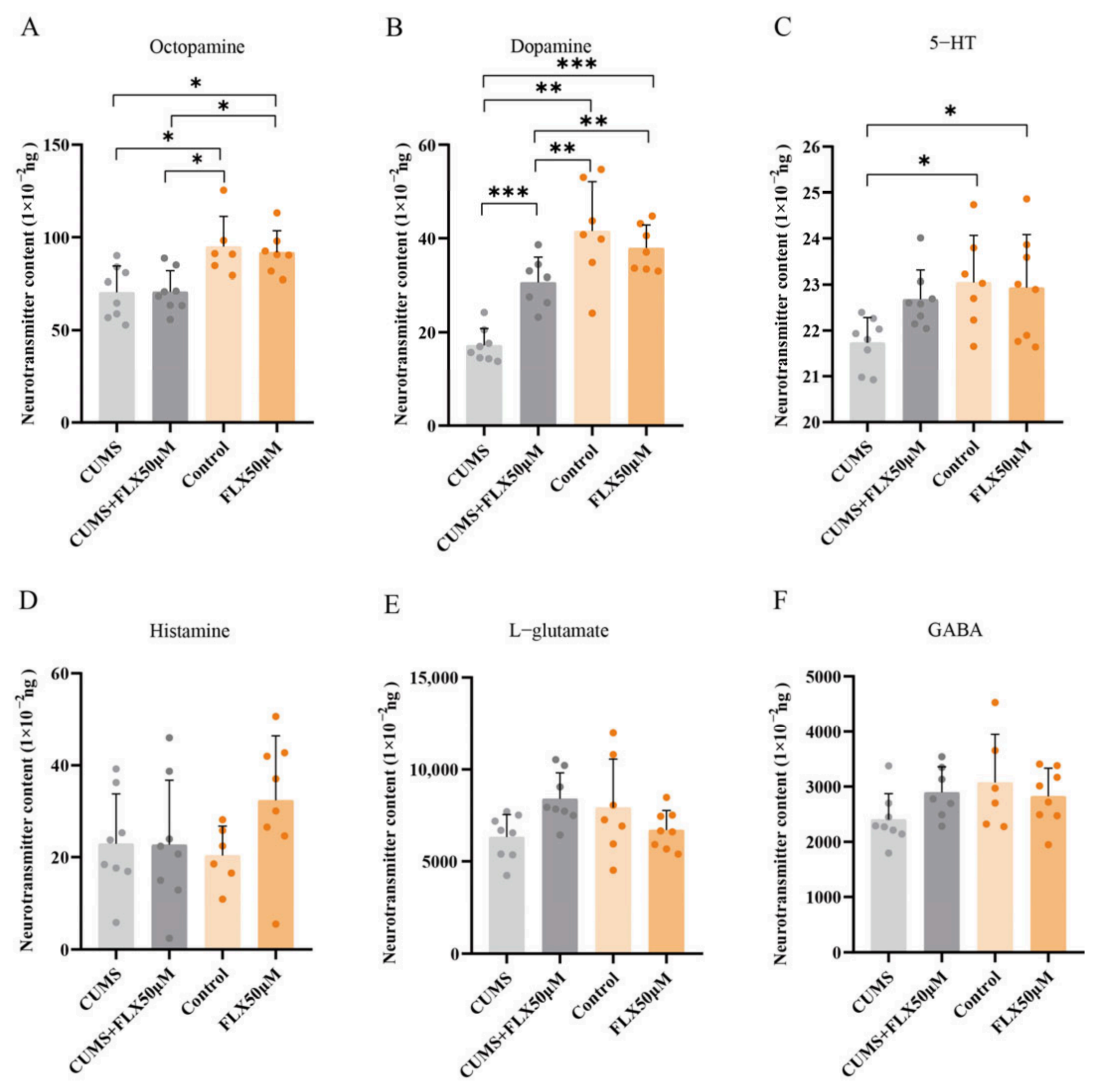
Brain neurotransmitter levels in honeybees under four treatments. Brain levels of (**A**) OA, (**B**) DA, (**C**) 5-HT, (**D**) HA, (**E**) L-Glu, and (**F**) GABA were measured in the CUMS, CUMS + FLX 50 μM, Control, and FLX 50 μM groups (*n* = 8 per group). Bars show mean ± SEM; dots indicate individual biological replicates. Significant comparisons were indicated by brackets: for OA, CUMS vs. Control, *t* = −2.96, df = 9.91, *p* < 0.05; CUMS + FLX 50 μM vs. Control, *t* = −3.13, df = 8.37, *p* < 0.05; CUMS vs. FLX 50 μM, *t* = −3.23, df = 12.98, *p* < 0.05; and CUMS + FLX 50 μM vs. FLX 50 μM, *t* = −3.57, df = 12.53, *p* < 0.05; for DA, Control vs. CUMS, *t* = 5.83, df = 7.22, *p* < 0.01; Control vs. CUMS + FLX 50 μM, *t* = 3.41, df = 8.85, *p* < 0.01; CUMS vs. CUMS + FLX 50 μM, *t* = −5.69, df = 10.38, *p* < 0.001; CUMS vs. FLX 50 μM, *t* = −9.20, df = 10.89, *p* < 0.001; and CUMS + FLX 50 μM vs. FLX 50 μM, *t* = −3.12, df = 11.94, *p* < 0.01; for 5-HT, Control vs. CUMS, *t* = 3.06, df = 8.96, *p* < 0.05, and CUMS vs. FLX 50 μM, *t* = −2.68, df = 10.05, *p* < 0.05. No significant pairwise differences were observed for HA, L-Glu, and GABA. * *p* < 0.05, ** *p* < 0.01, and *** *p* < 0.001. CUMS: chronic unpredictable mild stress; Control: no CUMS; CUMS + FLX 50 μM: CUMS with 50 μM fluoxetine treatment; FLX 50 μM: 50 μM fluoxetine treatment without CUMS. OA: octopamine; DA: dopamine; 5-HT: serotonin; HA: histamine; L-Glu: L-glutamate; GABA: γ-aminobutyric acid.

In this study, the levels of major brain neurotransmitters in honeybees were quantified following fluoxetine administration during CUMS exposure. Fluoxetine treatment during CUMS significantly increased dopamine levels in CUMS-exposed honeybees (Figure 6B, *t* = −5.69, df = 10.38, *p* < 0.001), whereas 5-HT levels showed a non-significant upward trend (Figure 6C, *p* > 0.05). Although dopamine levels were increased by fluoxetine, they remained significantly lower in the CUMS + FLX 50 μM group than in the Control (*t* = 3.41, df = 8.85, *p* < 0.01) and FLX 50 μM groups (*t* = −3.12, df = 11.94, *p* < 0.01) (Figure 6B). In contrast, 5-HT levels in the CUMS + FLX 50 μM group did not differ significantly from those in the Control group (Figure 6C, *p* > 0.05). Fluoxetine treatment during CUMS did not significantly alter octopamine levels, which remained significantly lower in the CUMS + FLX 50 μM group than in the Control (*t* = −3.13, df = 8.37, *p* < 0.05) and FLX 50 μM groups (*t* = −3.57, df = 12.53, *p* < 0.05) (Figure 6A). No significant effects of fluoxetine treatment during CUMS were detected on histamine, L-Glu, or GABA levels (Figure 6D–F, *p* > 0.05). Furthermore, fluoxetine treatment alone did not significantly affect any of the measured neurotransmitters in healthy honeybees compared with the Control group (Figure 6A–F, *p* > 0.05). These results suggest that fluoxetine partially ameliorated the CUMS-induced neurotransmitter disturbance, primarily by increasing dopamine levels, although dopamine was not fully restored to the Control level.

## 4. Discussion

The results indicated that honeybees exposed to the eight CUMS protocols for eight days exhibited significant depression-like behavioral alterations. In the spontaneous activity test, all CUMS groups showed varying degrees of reduced activity compared with the Control group (Figure S1 and Figure 2A,B), consistent with the decreased spontaneous locomotion observed in the open-field test of mouse depression models [37]. This shared behavioral phenotype may partly indicate a broad impairment of neuroplasticity in key brain regions induced by chronic stress. Comparisons among CUMS protocols that differed only in the intensity of a single stressor showed significant differences in SAT for some treatment pairs, whereas no significant differences were detected in SPT, SER, or PER (Figure 4A). In general, these behavioral outcomes did not display a clear intensity-dependent trend.

One possible explanation is that chronic stress may impair neuroplasticity in the honeybee brain, thereby limiting the adaptive remodeling of neural connections and circuit functions in response to changes in stressor intensity [38]. In depression models, impaired neuroplasticity in brain regions such as the hippocampus may reduce the ability of individuals to adapt to variations in stress intensity, resulting in behavioral responses that fail to exhibit a systematic intensity-dependent pattern [39,40]. After chronic stress treatment, depressive animal models exhibit deficits in hedonic behavior, which is one of the core symptoms of depression, namely anhedonia. In mice and fruit flies, this is manifested as a reduced preference for sucrose solutions [41,42,43]. In the present study, CUMS reduced the preference of honeybees for high-concentration sucrose solutions (Figure S2), indicating that CUMS induces a loss of reward responsiveness in honeybees, which was phenotypically consistent with the anhedonia observed in traditional animal models of depression. The PER of honeybees is an innate appetitive feeding response elicited by sucrose-related stimuli [44].

Defensive behavioral responses in animals can generally be divided into active and passive defense [45]. Active defense is characterized by escape and attack behaviors upon encountering threats, as well as exploratory behaviors in unfamiliar environments [46]. Passive defense is characterized by immobility and avoidance behaviors [47,48]. The stinging response of honeybees is an active defensive reaction to external threats, and the severity of the stinging response reflects the defensive level of honeybees [35,49,50]. Therefore, compared with the Control group, honeybees subjected to chronic stress exhibited stronger stinger extension scores (Figure S3), which was phenotypically consistent with the enhanced active defense observed in depressed mice [51] exposed to repeated social defeat stress. When honeybees perceive the odor of a sucrose solution or directly contact it, they instinctively extend their proboscis to ingest food [52]. This behavior demonstrates the innate motivation of honeybees to obtain food after receiving food-related cues. The PER latency of honeybees after CUMS exposure was significantly prolonged (Figure S4), indicating reduced responsiveness to food-related stimuli or feeding motivation, similar to the decreased feeding motivation observed in depressive mice [53].

Alterations in neurotransmitters provide important neurochemical evidence to assess whether chronic stress leads to depression-like physiological changes in honeybees. HA, OA, DA, 5-HT, GABA, and L-Glu are involved in regulating emotion-related behaviors, cognition, motivation, and stress responses [41,54,55]. In the present study, DA, 5-HT, and OA levels were significantly reduced to varying degrees in most CUMS treatment groups compared with the Control group (Figure 3). OA is a widely distributed monoamine neurotransmitter in insects with functions analogous to those of norepinephrine in mammals, including the regulation of behavior, energy metabolism, and stress responses [56,57,58,59]. In chronic stress models of *D. melanogaster*, significant reductions in dopamine, 5-HT, and octopamine levels have also been detected [60]. Alterations in L-Glu, HA, and GABA have likewise been associated with depression, although significant changes in these neurotransmitters were observed only in some CUMS treatment groups relative to the Control group (Figure 3). L-Glu is a major excitatory neurotransmitter in the central nervous system, and its accumulation in the hippocampus of depressive rats can induce neurotoxicity and apoptosis in neurons and astrocytes, thereby disrupting neural network function [61]. Reduced brain HA levels have also been reported in depressive mice, although its specific role in the pathogenesis of depression remains unclear [62]. Further comparisons between CUMS protocols differing only in the intensity of a single stressor revealed significant differences in HA, OA, DA, 5-HT, GABA, and L-Glu levels in some pairwise comparisons, but these changes did not follow a consistent intensity-dependent pattern (Figure 4B). These findings suggest that the neurochemical responses of honeybees to chronic combined stress are not determined solely by the intensity of an individual stressor; increasing the intensity of a single stressor does not necessarily exacerbate neurochemical abnormalities in honeybees [63].

Fluoxetine is widely applied as a classic antidepressant for treating depression. Therefore, fluoxetine administration was applied in this study to further assess whether behavioral and neurochemical abnormalities induced by CUMS in honeybees could be pharmacologically reversed. Spontaneous activity, sucrose preference, and defensive behavioral response were significantly improved after FLX administration in depressive-like honeybees, whereas no significant effect was found on PER latency (Figure 5A–E). Interestingly, sucrose preference and defensive behavioral response returned to baseline levels, whereas spontaneous locomotor activity did not fully recover (Figure 5A–C). This behavior-specific recovery is broadly consistent with earlier findings in *Drosophila*, where FLX reversed stress-induced impairments in sucrose preference and aggression-related behavior [60,64], but showed limited ability to restore spontaneous locomotor activity [32,34]. At the same time, FLX treatment showed a numerical tendency to shorten the prolonged PER latency induced by CUMS in honeybees, although no significant behavioral recovery was detected when compared with the sole CUMS group (Figure 5D,E). From the perspective of neural mechanisms, PER latency reflects the temporal processing from antennal sucrose detection to appetitive motor output in honeybees [65]. This reflex largely depends on intact neurotransmitter signaling [66], whereas FLX significantly increased DA levels, produced a nonsignificant increase in 5-HT levels, and had no significant effect on OA levels (Figure 6A–C). OA regulates sucrose reward processing, appetitive PER, and foraging-related responses in honeybees [67,68]. In contrast, 5-HT suppresses the PER and reduces appetitive reward-related responsiveness, suggesting that imbalance between OA and 5-HT in the brain may interfere with PER in honeybees [69]. Nevertheless, FLX treatment failed to restore the balance between OA and 5-HT, and therefore was unable to reverse the prolonged PER latency. In addition, in *Drosophila*, FLX is used to treat depression-like states; it restores climbing motivation and sucrose preference, reduces immobility and aggressive behavior, and increases brain DA and 5-HT levels [32,64,69,70,71]. Collectively, DA and 5-HT may act as key neurochemical biomarkers involved in stress-induced depression-like behaviors in honeybees (Figure 6B,C).

The depression-like behavior induced by CUMS in honeybees still requires further investigation, which will be addressed in future work. In addition, several limitations exist in this study. First, although stressors were randomly selected during the CUMS procedure, starvation stress was not applied on the day before SPT and PER in order to reduce its potential influence on food-related behavioral assays. Because feeding condition may affect sucrose preference and proboscis extension responses [72,73], the possible direct impact of acute starvation on these behavioral assays was minimized, and short-term food deprivation has also been used as a mild stressor in CUMS paradigms [74]. Nevertheless, the effect of stressor sequence cannot be fully excluded. Second, repeated or prolonged stress exposure may lead to the development of stress-avoidance coping strategies in animals. Moreover, whether the honeybee brain is capable of higher-order cognitive behaviors related to thoughts and emotions remains to be clarified. Despite these limitations, the present study improved the success rate of establishing depression-like behavior models in honeybees, which will greatly facilitate future research on the pathogenesis of depression, as well as the screening, efficacy evaluation, and mechanistic investigation of antidepressant drugs.

## Data Availability

All study data are included in the article and Supplementary Material.

## Supplementary Materials

The following supporting information can be downloaded at: https://www.mdpi.com/article/10.3390/insects17080773/s1, Figure S1: Spontaneous activity of bees within 2 min; Figure S2: Experimental results of sucrose preference in bees; Figure S3: Sting extension score; Figure S4: Proboscis extension reaction time; Figure S5: Survival curves of honeybees under different stress regimens; Table S1: Protocol for stress treatment in honeybees; Table S2: Standard curve equations of the standards for each substance.
