# Supplementary material for: Establishment and Validation of a CUMS Model for Depression-like Behavior in Honeybees: A Tool for Pollinator Stress Research
Source: Insects. 2026 Jul 27;17(8):773. doi: 10.3390/insects17080773 (PMC13513438; doi:10.3390/insects17080773)
Supplement: Supplementary file 1 [file insects-17-00773-s001.zip › insects-4381827-supplementary.pdf]

Supplementary information

Figures S1–S5

Tables S1-S2

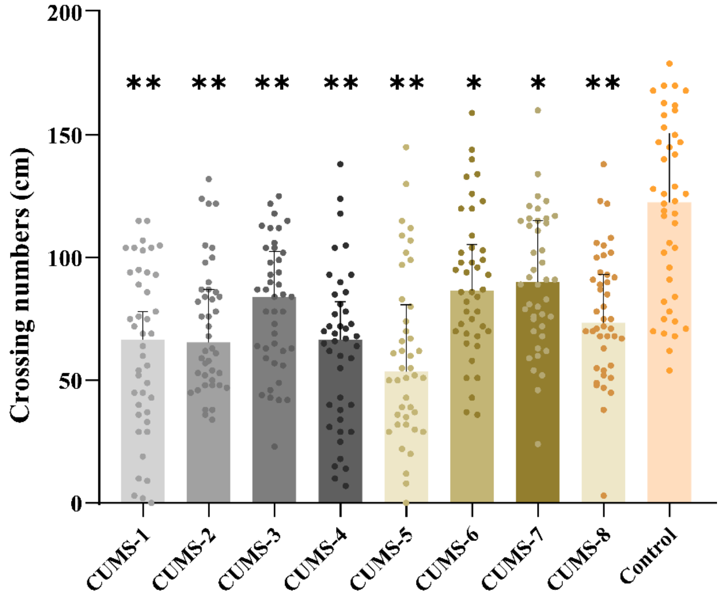

Figure S1. Spontaneous activity of bees within 2 min. All CUMS-treated groups displayed a significant reduction in grid crossings relative to the Control group (n = 42 per group). Welch's one-way ANOVA confirmed significant overall differences across groups ($F(8, 153.61) = 12.64$, $P < 0.01$). Post-hoc pairwise comparisons against the Control group were performed using Welch's t-tests with Holm correction: CUMS-1 vs Control, $t = -7.44$, df = 81.66, $P < 0.01$; CUMS-2 vs Control, $t = -6.98$, df = 74.65, $P < 0.01$; CUMS-3 vs Control, $t = -5.74$, df = 74.12, $P < 0.01$; CUMS-4 vs Control, $t = -7.68$, df = 80.55, $P < 0.01$; CUMS-5 vs Control, $t = -7.82$, df = 81.73, $P < 0.01$; CUMS-6 vs Control, $t = -4.15$, df = 78.75, $P < 0.05$; CUMS-7 vs Control, $t = -4.15$, df = 76.51, $P < 0.05$; CUMS-8 vs Control, $t = -6.27$, df = 74.37, $P < 0.01$. No clear trend correlating stress intensity with activity levels was identified among the CUMS subgroups. Data are shown as mean ± SEM, with individual honeybees represented by scattered dots. $P < 0.01$ denotes a significant difference compared with the Control group.

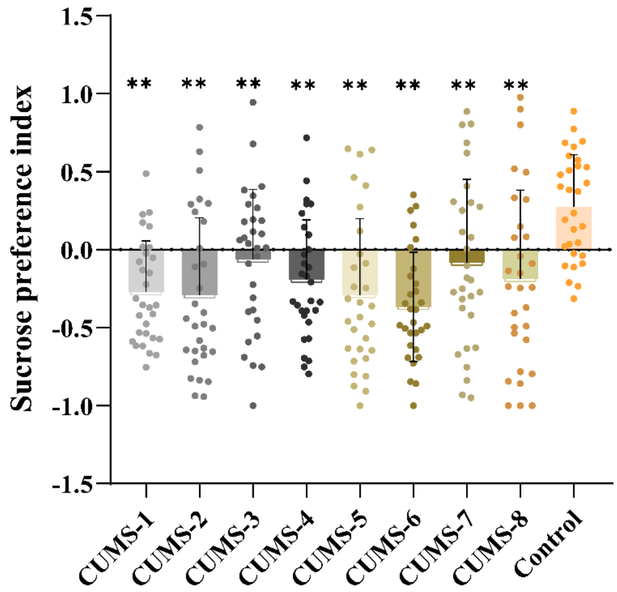

Figure S2. Experimental results of sucrose preference in bees. All CUMS-exposed groups

exhibited a marked reduction in sucrose preference index relative to the Control group (n = 30 per group). Welch's one-way ANOVA detected significant overall intergroup differences (F(8, 108.49) = 8.41, *P* values less than 0.01). Post-hoc pairwise comparisons against the Control group were performed using Welch's t-tests with Holm correction: CUMS-1 vs Control, t = −6.36, df = 57.97, *P* < 0.01; CUMS-2 vs Control, t = −5.17, df = 50.92, *P* < 0.01; CUMS-3 vs Control, t = −3.30, df = 53.70, *P* < 0.01; CUMS-4 vs Control, t = −5.00, df = 57.06, *P* < 0.01; CUMS-5 vs Control, t = −5.21, df = 51.41, *P* < 0.01; CUMS-6 vs Control, t = −7.22, df = 57.91, *P* < 0.01; CUMS-7 vs Control, t = −3.10, df = 48.81, *P* < 0.01; and CUMS-8 vs Control, t = −3.82, df = 46.98, *P* < 0.01. Data are shown as mean ± SEM, with individual honeybees represented by scattered dots. *P* values less than 0.01 denote statistically significant differences compared with the Control group.

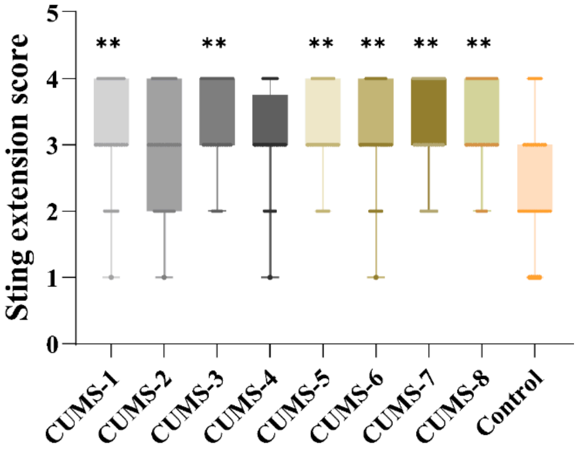

Figure S3. Sting extension score. All CUMS-exposed groups exhibited elevated sting extension scores relative to the Control group. Kruskal–Wallis test identified significant intergroup differences (*H* = 25.53, *P* = 0.0013). Post-hoc Mann–Whitney U tests with Holm correction were used for comparisons versus the Control group: CUMS-1 vs Control, *W* = 1218.5, *P* < 0.01; CUMS-2 vs Control, *W* = 1156.0, *P* > 0.05; CUMS-3 vs Control, *W* = 1311.5, *P* < 0.01; CUMS-4 vs Control, *W* = 1113.0, *P* > 0.05; CUMS-5 vs Control, *W* = 1235.0, *P* < 0.01; CUMS-6 vs Control, *W* = 1199.5, *P* < 0.01; CUMS-7 vs Control, *W* = 1249.0, *P* < 0.01; and CUMS-8 vs Control, *W* = 1263.5, *P* < 0.01. Boxplots present medians and interquartile ranges. *P* values less than 0.01 indicate statistically significant differences compared with the Control group.

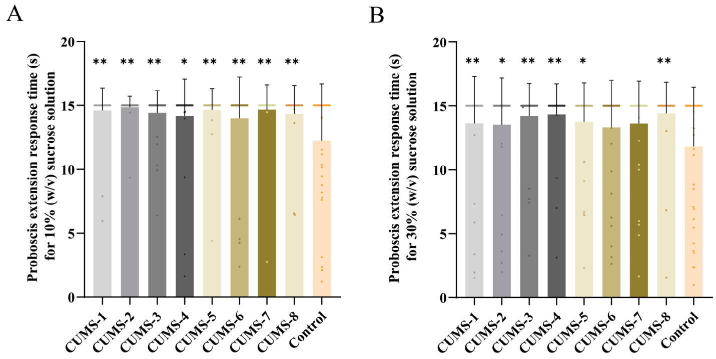

Figure S4. Proboscis extension reaction time. (A) PER latency upon 10% (w/v) sucrose stimulation. All CUMS-exposed groups had prolonged PER latency versus Control (n = 42; n = 40 for CUMS-4 and CUMS-7). Kruskal–Wallis test indicated significant intergroup differences (H = 31.32, P < 0.01). Post-hoc Mann–Whitney U tests with Holm correction for comparisons with Control: CUMS-1 vs Control, U = 1152.0, P < 0.01; CUMS-2 vs Control, U = 1164.0, P < 0.01; CUMS-3 vs Control, U = 1108.5, P < 0.01; CUMS-4 vs Control, U = 1028.0, P < 0.05; CUMS-5 vs Control, U = 1144.5, P < 0.01; CUMS-6 vs Control, U = 1102.0, P < 0.01; CUMS-7 vs Control, U = 1102.0, P < 0.01; CUMS-8 vs Control, U = 1098.5, P < 0.01 (B) PER latency upon 30% (w/v) sucrose stimulation. Kruskal–Wallis test revealed significant overall group differences (H = 24.04, P = 0.0023). PER latency increased significantly in CUMS-1, CUMS-2, CUMS-3, CUMS-4, CUMS-5 and CUMS-8, whereas CUMS-6 and CUMS-7 showed no difference from Control. Post-hoc Mann–Whitney U tests with Holm correction for comparisons with Control: CUMS-1 vs Control, U = 1116.5, P < 0.01; CUMS-2 vs Control, U = 1100.0, P < 0.05; CUMS-3 vs Control, U = 1162.0, P < 0.01; CUMS-4 vs Control, U = 1121.0, P < 0.01 CUMS-5 vs Control, U = 1111.0, P < 0.05; CUMS-6 vs Control, U = 1068.0, P > 0.05; CUMS-7 vs Control, U = 1036.0, P > 0.05; CUMS-8 vs Control, U = 1194.0, P < 0.01.

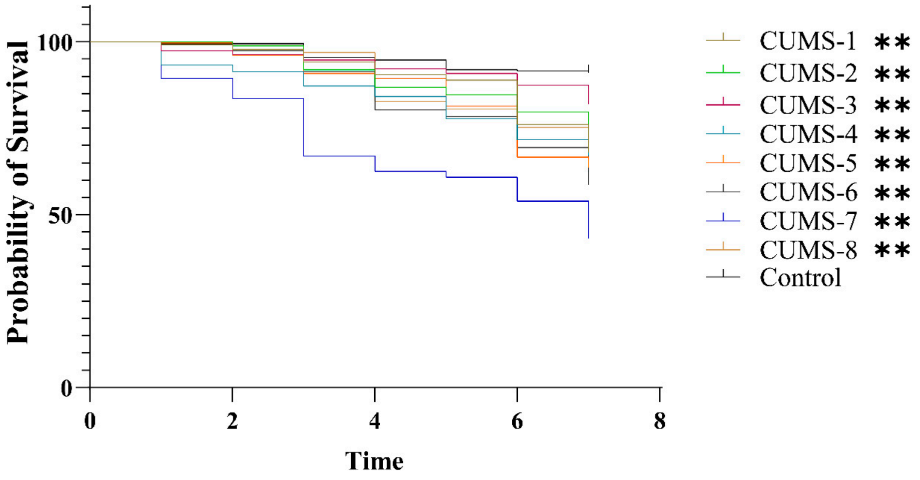

Figure S5. Survival curves of honeybees under different stress regimens. Survival curves were estimated using the Kaplan–Meier method (Control and CUMS-1 to CUMS-8, n = 360). The overall log-rank test revealed significant differences among the nine groups (χ^2 = 301.64, df = 8, P < 0.01). Pairwise log-rank tests with Holm correction for comparisons with Control: CUMS-1 vs Control, χ^2 = 57.95, P < 0.01; CUMS-2 vs Control, χ^2 = 38.65, P < 0.01; CUMS-3 vs Control, χ^2 = 14.18, P < 0.01; CUMS-4 vs Control, χ^2 = 64.05, P < 0.01; CUMS-5 vs Control, χ^2 = 77.34, P < 0.01; CUMS-6 vs Control, χ^2 = 100.46, P < 0.01; CUMS-7 vs Control, χ^2 = 196.93, P < 0.01; and CUMS-8 vs Control, χ^2 = 28.95, P < 0.01. Among the CUMS groups, CUMS-3 showed the highest Day-7 survival rate (81.94%), whereas CUMS-7 showed the lowest survival rate (43.06%). ** indicates a significant difference compared with the Control group (P < 0.01).

Table S1. Protocol for stress treatment in honeybees.

CUMS Protocol	Odor Stress	Shock Stress	Electric Stress	Starvation Stress
CUMS-1	8%	200HZ	5v	2h
CUMS-2	8%	200HZ	7v	2h
CUMS-3	24%	200HZ	5v	2h
CUMS-4	24%	200HZ	7v	2h
CUMS-5	8%	200HZ	5v	4h
CUMS-6	8%	200HZ	7v	4h
CUMS-7	24%	200HZ	5v	4h
CUMS-8	24%	200HZ	7v	4h

Table S2. Standard curve equations of the standards for each substance.

Standard	Standard Curve Equation	R^2
Dopamine (DA)	y=335.9x-2553	0.9947
Octopamine (OA)	y=503.78x-10055	0.9966
5-HT	y=2321.4x-122314	0.9914
Histamine (HA)	y=4237.9x+155547	0.9959
L-glutamate (L-Glu)	y=274.65x+16431	0.9993
GABA	y=358.82x+48168	0.9983
